# Notes and Notices

**Published:** 1883-02

**Authors:** 


### NOTES AND NOTICES.

REMOVALS.—Dr. E. S. Breyfogle has removed from San Jose to San Francisco, and has located at 14 Dupont Street, between Geary and Market.

Dr. C. P. Schlick, from New York to Brooklyn.

Dr. C. T. Canfield, from Indianapolis to Chicago, 244 Lincoln Avenue.

NEW YORK STATE HOMŒOPATHIC SOCIETY.—The Thirty-second Annual Meeting of the Homœopathic Medical Society, of the State of New York, will be held in the Court of Appeals Room, New Capitol (north entrance), Albany, on Tuesday and Wednesday, February 13th and 14th, 1883. The session will open at 10 A. M. of the first day, and the annual address will be delivered by the President, Dr. Jno. J. Mitchell, of Newburgh, in the Assembly Chamber, New Capitol, on Tuesday evening.

HAVANA, N. Y., *Jan. 12th, 1883.* A. P. HOLLITT, *Secretary.*

NEW JOURNAL.—Dr. Wm. Bœricke commenced in November last to publish the *California Homœopath*, "a bi-monthly devoted to the interests of Homœopathy on the Pacific Coast." The first number, a bright and interesting sheet, gives promise of the good things to come. We bespeak for our young brother a cordial welcome, and hope it may do good work in its mission on the far Pacific coast.

THE U. S. MEDICAL INVESTIGATOR is now a weekly journal. We wish the *Investigator* success in its new venture.

THE LOVE PARASITE.—A California physician who discovered a new disease—love madness—has been experimenting with the persons afflicted therewith and has produced the "love parasite," or bacillus micrococus. This he cultivated up to the twentieth generation, and with the parasites of that generation he inoculated a number of subjects. The inoculation was invariably successful, symptoms of the disease appearing in a very short time after the operation. A bachelor, aged fifty, on the first day after inoculation had his whiskers dyed, ordered a suit of new clothes and a set of false teeth, bought a top buggy, a bottle of hair restorer, a diamond ring and a guitar, and began reading Byron's poems. The inoculation produced symptoms of the same nature in a young lady of forty-five.—*Ext.*

